# Insights on the Formulation of Herbal Beverages with Medicinal Claims According with Their Antioxidant Properties

**DOI:** 10.3390/molecules18032851

**Published:** 2013-03-04

**Authors:** João C. M. Barreira, Ana L. Morais, Isabel C. F. R. Ferreira, M. Beatriz P. P. Oliveira

**Affiliations:** 1 REQUIMTE Chemistry and Technology Network/Department of Chemical Sciences, Faculty of Pharmacy, University of Porto, Rua Jorge Viterbo Ferreira, 228, 4050-313 Porto, Portugal; E-Mails: jbarreira@ipb.pt (J.C.M.B.); aluisa.morais@gmail.com (A.L.M.); 2 Mountain Research Center, School of Agriculture, Polytechnic Institute of Bragança, Campus de Santa Apolónia, 1172, 5301-855 Bragança, Portugal

**Keywords:** antioxidant activity, biomolecules, herbal drinks, optimized formulations

## Abstract

Several herbal beverages claim medicinal benefits due to their antioxidant properties. However, operational factors such as the extracted herbal component, preparation method or concentration levels, might influence their biological activity. To assess this effect, the antioxidant activity of beverages prepared with *Camellia sinensis*, *Aspalathus linearis* or *Cochlospermum angolensis*, used solely or mixed with different fruit, plant or algae extracts, was studied using different formulations (bags, leaves, roots, granulates, powders, liquids) and different preparation methods (infusion, solubilisation or promptly used). The DF_50_ (dilution factor responsible for 50% of antioxidant activity) values were calculated to compare their antioxidant activity. A linear discriminant analysis was used to categorize the assayed samples according to their antioxidant activity and bioactive molecules profiles. The results indicated that antioxidant activity and antioxidant compounds are significantly affected by formulation and preparation method, but overall the labelled antioxidant benefits were validated. Green tea showed the highest activity, but with different behaviour within each used formulation. The high DF_50_ values calculated for some products might be used to adjust the dietary dose or formulation, preventing also putative pro-oxidant effects. Hence, the obtained results might be useful to define the formulation of these highly consumed herbal beverages, enhancing their health effects.

## 1. Introduction

Under stress, living organisms produce more reactive oxygen species (ROS, e.g., superoxide anion radicals, hydroxyl radicals and hydrogen peroxide) than enzymatic (e.g., superoxide dismutase, glutathione peroxidase, and catalase) and non-enzymatic (e.g., ascorbic acid, α-tocopherol, glutathione, carotenoids, and flavonoids) antioxidants, causing an imbalance that may lead to cell damage and health problems [1]. Supplementing the diet with antioxidant compounds contained in natural plant sources can help solve this problem. In fact, natural antioxidants might have an important function in preventive medicine approaches [2].

Despite the recognized action of phenolic compounds as health-promoting components [3,4,5], especially due to their antioxidant and antiradical potentials [6,7,8], there are several reports highlighting them as being strong astringents, thus posing a potential problem for manufacturers, who develop products rich in polyphenolic antioxidants [9,10]. One solution to this problem is attempting to mask the astringency of these compounds with specific food supplements like polysaccharides [11]. On the other hand, astringency is directly associated with the concentration of phenolic compounds. Thereby, lowering the phenolic compound concentration in herbal beverages, while ensuring that their antioxidant activity is retained, may offer the possibility of obtaining well-accepted products without affecting their health-promoting qualities.

Tea, a product made from leaf and bud of the plant *Camellia sinensis*, is, following water, the most consumed beverage in the World. It can be categorized into green tea (non-fermented; produced by drying and steaming the fresh leaves to inactivate the polyphenol oxidase, preventing oxidation processes), black and red teas (fermented; undergo a post-harvest fermentation stage before drying and steaming, which generates the oligomeric polyphenolic compounds theaflavins and thearubigins; participation of polyphenol oxidase in black tea, and microorganisms in red tea). African Rooibos (*Aspalathus linearis*) and Borututu (*Cochlospermum angolensis*) tisanes are also highly consumed [12,13,14].

When consumed within a balanced diet, these herbal beverages may have the capacity to improve the antioxidant status, preventing oxidative damage in humans [13], reducing cardiovascular disease and some forms of cancer, exerting a neuroprotective effect, as well as preventing other disorders related to oxidative stress [13,15], including the control of cellular redox dysfunction linked to type 2 diabetes [16]. These functions have been attributed to their high content of polyphenols (catechins or flavan-3-ols, theaflavins, thearubigins and proanthocyanidins) [17]. Epidemiological studies suggested that tea polyphenols are effective for prevention of cancer and cardiovascular diseases [18]. However, it has been shown that some green tea extracts [19] and flavonoid-enriched fractions of rooibos can have pro-oxidant activities. In fact, regarding health promotion, one should bear in mind that potent antioxidants can also display pro-oxidant activities, leading to oxidative damage of cellular components [20].

Green, black and red teas are commercially available in different formulations (bags, leaves, roots, granulates, powders, liquids) and can be prepared by infusion, solubilisation or be drunk directly. The used formulation or preparation method may greatly influence the antioxidant activity as it was reported for the free radical scavenging activity of leaf extracts, tea liquor and their combination with dietary supplements [21]. In the present work, a comparative study of antioxidant activity and main antioxidant compounds of highly consumed herbal beverages was performed. Besides finding the most effective formulations, it was also intended to verify if the doses recommended in labels are adequate to guarantee the aimed antioxidant effects.

## 2. Results and Discussion

### 2.1. Biomolecules with Antioxidant Activity

In order to obtain a comprehensive knowledge about the effect of diluting the herbal beverage concentration, the complex chemical composition of natural matrices should be considered. The individual compounds present in these matrices might act in a synergistic, additive or antagonist way [22]. To overcome this limitation, different *in vitro* test systems were applied: reducing power, scavenging capacity against 2,2-diphenyl-1-picrylhydrazyl radical (DPPH), β-carotene bleaching inhibition against linoleate radicals and lipid peroxidation inhibition using thiobarbituric acid reactive substances (TBARS). TBARS is a more reliable method because the test sample needs to primarily permeate inside the biological cells to elicit its antioxidant action. In this way, the biological stability of the test sample is evaluated in addition to its antioxidant action [23], but considering that the total antioxidant capacity is dependent on several factors, so a “battery” of assays measuring different aspects of the behaviour of antioxidants is strongly recommended to generate a complete antioxidant profile [24].

Table 1 gives the results obtained for the dilution factor from “tea” stock solution corresponding to 50% of antioxidant activity (DF_50_) expressed with the decimal numbers allowed by the standard deviation. The DF_50_ values were chosen instead of the commonly used EC_50_ (effective concentrations) values, since those are a more realistic antioxidant activity measure on view of consumer’s habits.

According to the information available in labels (formulation, preparation method and amount used), the final concentration of each “tea” is different. Therefore it is useful to understand if those recommendations correspond to the most appropriate antioxidant properties, or if a specific dilution would be more adequate.

In general, the solubilised beverages (S) proved to have higher antioxidant activity; nevertheless, the maximal DF_50_ for DPPH radical scavenging capacity (D-Gt/E), reducing power (D-Gt/E) and inhibition of lipid peroxidation using β-carotene-linoleate model (D-GtL/L) were obtained in ready to use (D) formulations; while the lipid peroxidation in brain homogenates was better inhibited by green tea infusion (I-Gt/B). The lowest antioxidant activity was obtained for borututu tea, which was prepared with *C. angolensis* roots. The chosen preparation method was previously reported as affecting greatly the antioxidant activity of “teas” [25].

Ascorbic acid was the main antioxidant compound found in all the studied “teas” (Table 1). In fact, this vitamin is commonly added to tea formulations [26] to stabilize the tea catechins (the most important phenolic compounds present in tea) in the intestine, where the pH is neutral or alkaline, before absorption [27].

**Table 1:** Antioxidant activity (df_50_ values; mg/mL) and biomolecules present in the “teas”. The results are expressed as mean ± sd (n = 9).

Samples	DPPH scavenging activity ^a^	Reducing power ^a^	β-Carotene bleaching inhibition ^a^	TBARS inhibition ^a^	Phenolics (mg ClAE/mL) ^a^	Flavonoids (mg CE/mL) ^a^	Flavonols (mg QE/mL) ^a^	Tartaric esters (mg CAE/mL) ^a^	Ascorbic acid (mg/mL) ^a^
I-Gt/B	3.01 ± 0.16 h	31.2 ± 0.2 g	1.9 ± 0.3 h	203 ± 12 a	0.74 ± 0.05 e	0.127 ± 0.003 d	0.020 ± 0.001 g	0.038 ± 0.002 cde	7.0 ± 0.2 a
I-GtH/B	56 ± 4 b	118.0 ± 0.4 b	11.7 ± 0.5 ef	143 ± 17 b	1.96 ± 0.02 a	0.25 ± 0.01 b	0.077 ± 0.001 b	0.086 ± 0.003 a	0.249 ± 0.001 gh
I-GtL/B	3 ± 1 h	22.9 ± 0.4 i	2.8 ± 0.4 gh	83 ± 6 ef	0.77 ± 0.01 e	0.109 ± 0.004 e	0.047 ± 0.001 d	0.042 ± 0.002 cd	2.20 ± 0.03 c
I-Gt/Lv	19.2 ± 0.4 fg	25.5 ± 0.2 h	2.7 ± 0.2 gh	76 ± 1 f	0.96 ± 0.02 d	0.06 ± 0.02 f	0.020 ± 0.002 g	0.011 ± 0.001 i	0.77 ± 0.04 d
I-Rt/B	2.0 ± 0.3 h	12 ± 2 k	2.0 ± 0.4 h	16 ± 2 hi	0.56 ± 0.02 g	0.164 ± 0.005 c	0.110 ± 0.004 a	0.077 ± 0.004 b	0.08 ± 0.01 ij
I-Bt/R	1.6 ± 0.2 ^b^	1.016 ± 0.004 p	1.3 ± 0.2 h	1.5 ± 0.1 i	0.024 ± 0.002 j	0.015 ± 0.001 h	0.005 ± 0.001 k	0.005 ± 0.002 j	0.12 ± 0.02 ij
S-Gt/G1	36 ± 5 d	47.3 ± 0.3 d	45 ± 8 b	133 ± 25 bcd	0.6 ± 0.2 g	0.060 ± 0.001 f	0.022 ± 0.001 g	0.03 ± 0.01 defg	0.396 ± 0.002 ef
S-Gt/G2	24 ± 1 e	39.9 ± 0.1 e	3.7 ± 0.4 gh	123 ± 10 cd	0.73 ± 0.02 e	0.058 ± 0.005 f	0.041 ± 0.004 e	0.036 ± 0.004 cdef	0.32 ± 0.01 fg
S-Gt/P1	27 ± 1 e	40.9 ± 0.2 e	20 ± 2 d	137 ± 16 bc	0.63 ± 0.01 fg	0.55 ± 0.01 a	0.030 ± 0.002 f	0.031 ± 0.003 efg	0.434 ± 0.005 e
S-Gt/P2	20 ± 5 f	35 ± 1 f	8 ± 1 fg	144 ± 12 b	0.40 ± 0.01 h	0.155 ± 0.005 c	0.019 ± 0.001 gh	0.019 ± 0.002 hi	0.480 ± 0.003 e
D-Gt/E	149 ± 5 a	197.1 ± 0.5 a	12 ± 1 ef	118 ± 3 d	0.64 ± 0.02 fg	0.14 ± 0.01 d	0.053 ± 0.002 c	0.043 ± 0.003 c	0.047 ± 0.001 j
D-GtH/L	52 ± 2 c	6.64 ± 0.02 m	36 ± 5 c	73 ± 15 f	1.52 ± 0.05 b	0.155 ± 0.005 c	0.014 ± 0.001 i	0.033 ± 0.001 efg	2.88 ± 0.02 b
D-GtL/L	15 ± 1 g	21.7 ± 0.2 j	59 ± 4 a	100 ± 17 e	1.02 ± 0.01 d	0.028 ± 0.001 gh	0.015 ± 0.001 hi	0.028 ± 0.001 fg	0.13 ± 0.01 ij
D-Ga/L	1.89 ± 0.03 h	57 ± 1 c	2.6 ± 0.2 gh	53 ± 2 g	0.41 ± 0.02 h	0.127 ± 0.003 d	0.018 ± 0.001 gh	0.015 ± 0.003 i	2.87 ± 0.1 b
D-PRb/L	1.89 ± 0.03 h	8.70 ± 0.04 l l	35 ± 2 c	30 ± 1 h	1.44 ± 0.02 b	0.032 ± 0.002 g	0.022 ± 0.001 g	0.025 ± 0.002 gh	0.43 ± 0.01 e
D-Rt/L	3.38 ± 0.67 h	5.1 ± 0.1 n	13 ± 1 e	17 ± 2 hi	0.20 ± 0.01 i	0.032 ± 0.001 g	0.019 ± 0.001 gh	0.027 ± 0.002 fgh	0.14 ± 0.01 ij
D-Bt/L	36.8 ± 0.4 d	11.3 ± 0.1 k	13.8 ± 0.4 e	21 ± 4 h	1.11 ± 0.02 c	0.06 ± 0.02 f	0.03 ± 0.01 f	0.035 ± 0.002 cdef	0.31 ± 0.04 fg
D-LbLC/L	1.25 ± 0.05 h	2.34 ± 0.01 o	33 ± 8 c	30 ± 4 h	0.71 ± 0.02 ef	0.05 ± 0.01 f	0.009 ± 0.001 j	0.016 ± 0.003 i	0.160 ± 0.002 hi

^a^ In each column different letters mean significant differences (*p <* 0.05); ^b^ DF_25_.

The antioxidant compounds contents showed significant differences among the assayed “teas”. Phenolics (I-GtH/B), flavonols (I-Rt/B), tartaric esters (I-GtH/B) and ascorbic acid (I-Gt/B) were higher in infused samples. The lack of significant correlations between the quantified antioxidants and the DF_50_ values may indicate that other compounds (e.g., reducing sugars) might also be present, contributing to the measured antioxidant potential of the “teas”.

### 2.2. Linear Discriminant Analysis

**Figure 1:**
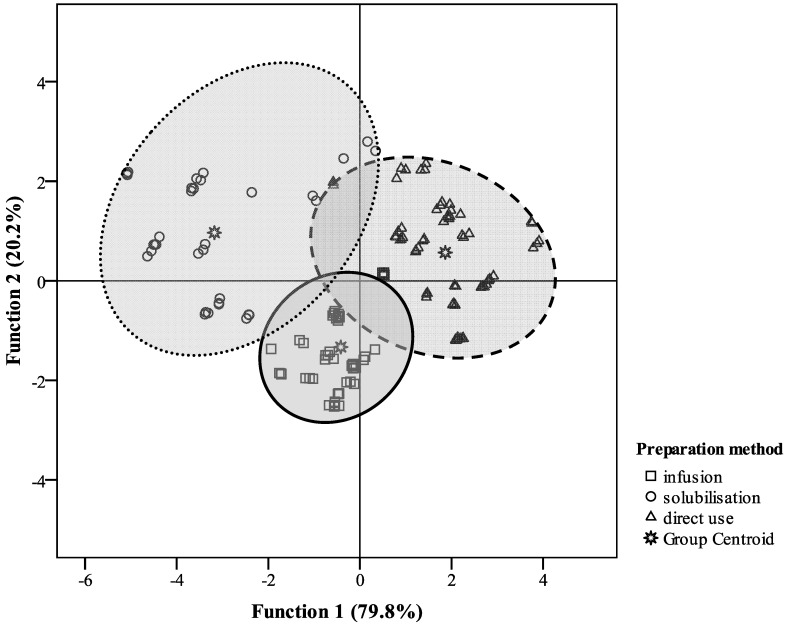
All-groups scatter plot for linear discriminant analysis of different “tea” formulations according with antioxidant activity and bioactive compounds contents.

The differences among formulations were highlighted in the performed stepwise LDA, which resulted in a discriminant model with two significant (*p* < 0.001 for the Wilks’λ test) discriminant functions (Figure 1). These two functions explained 100.0% of the observed variance. The first function mainly separates solubilisations from infusions (means of the canonical variance (MCV): infusions −0.415; solubilisations −3.176; direct uses 1.865), and was more correlated with TBARS inhibition and flavonoids. The second function improved the separation of direct uses from the remaining preparation methods (MCV: infusions −1.331; solubilisations −0.967; direct uses 0.568) being more correlated with β-carotene bleaching inhibition and flavonols. The analysed variables proved to have discriminant power, once that 90.3% of the original grouped cases as well as of the cross-validated groups were correctly classified.

Concerning the different formulation/preparation methods of each herbal beverage, green tea liquid extract proved to have higher antioxidant activity than the same tea prepared after infusion for all tested assays (except TBARS inhibition). A similar result was observed for green tea supplemented with lemon, which was more effective when readily used in comparison with its infusion for all the assays (except inhibition of lipid peroxidation using β-carotene-linoleate model). For the green tea supplemented with hibiscus, the infusion showed better results, with higher antioxidant activity for all assays except for inhibition of lipid peroxidation using β-carotene-linoleate model. Curiously, in the case of red tea with rooibos only the differences observed for reducing power and inhibition of lipid peroxidation using β-carotene-linoleate model were statistically significant. The higher antioxidant of green tea, as well as the influence of manufacturing processes on the properties of tea, are in accordance with a previous report [28].

## 3. Experimental

### 3.1. Standards and Reagents

The standards of Trolox (6-hydroxy-2,5,7,8-tetramethylchroman-2-carboxylic acid), L-ascorbic acid, caffeic acid, chlorogenic acid, malvidin 3-glucoside, and quercetin dehydrate were purchased from Sigma (St. Louis, MO, USA). 2,2-Diphenyl-1-picrylhydrazyl (DPPH) was obtained from Alfa Aesar (Ward Hill, MA, USA). All other chemicals and solvents were of analytical grade and purchased from common sources. Water was treated in a Milli-Q water purification system (TGI Pure Water Systems, Greenville, SC, USA).

### 3.2. Samples and Samples Preparation

Different herbal drinks, herein designated as “teas”, with medicinal indications (antioxidant effects, slimming, anti-carcinogenic, anti-aging, and nervous system stimulation) mentioned in their labels, were obtained from local markets. Samples were selected considering their availability in different commercial presentations: bags (B), leaves (Lv), roots (R), granulates (G), powders (P), and liquid extracts (E), in order to compare differences associated with the preparation method. Furthermore, samples were selected to check the differences among formulations including a single plant (*Camellia sinensis*, *Aspalathus linearis* or *Cochlospermum angolensis*) or mixtures composed by different fruits, plants or algae extracts. A stock-solution was prepared in each case according to labelled recommendations, either by infusion (I), solubilisation (S) or direct use (D), as described in Table 2. Several dilutions (1:10, 1:25, 1:50, 1:100, 1:200 and 1:1,000), were prepared and assayed for their antioxidant activity. Table 2 shows the alphanumeric codes that identify the samples used in this study. Three main groups were delineated according to the preparation method (I, S or D) and commercial presentation (B, Lv, R, G, P or E).

**Table 2:** Identification and preparation of the “teas” (stock solutions).

	Designation/formulation	Amount used ^a^	Time, Temperature ^a^	Code
Infusion	Green tea (*Camellia sinensis*)/Bag	1 bag (2.0 g) in 200 mL	5 min, 75 °C	I-Gt/B
	Green tea, pineapple, Hibiscus/Bag	1 bag (2.3 g) in 200 mL	5 min, 75 °C	I-GtH/B
	Green tea, lemon herb, algae/Bag	1 bag (1.5 g) in 200 mL	3 min, 100 °C	I-GtL/B
	Green tea/Leaves	1.62 g of leaves in 200 mL	3 min, 75 °C	I-Gt/Lv
	Rooibos red tea (*Aspalathus linearis*)/Bag	1 bag (1.5 g) in 200 mL	4 min, 100 °C	I-Rt/B
	Borututu tea (*Cochlospermum angolensis*)/Roots	0.27 g of roots in 200 mL	5 min, 75 °C	I-Bt/R
Solubilisation	Green tea, vitamin C/Granulate	4–5 teaspoons (16.55 g) in 200 mL	Direct, 75 °C	S-Gt/G1
			Direct, Room temperature (R_t_)	S-Gt/G2
	Green tea, vitamin C, red fruits/Powder	5.0 g of powder in 200 mL	Direct, 75 °C	S-Gt/P1
			Direct, R_t_	S-Gt/P2
Direct use	Green tea/Liquid extract	Direct (3 teaspoons- 30 mL)	Direct, R_t_	D-Gt/E
	Green tea, pineapple, Hibiscus/Liquid	Direct (Bottle of 250 mL)	Direct, R_t_	D-GtH/L
	Green tea, lemon/Liquid	Direct (Bottle of 330 mL)	Direct, R_t_	D-GtL/L
	Green apples, lemon, *Gingko biloba*/Liquid	Direct (Bottle of 250 mL)	Direct, R_t_	D-Ga/L
	Pomegranate, red berries/Liquid	Direct (Bottle of 330 mL)	Direct, R_t_	D-PRb/L
	Rooibos Red tea/Liquid	Direct (Bottle of 330 mL)	Direct, R_t_	D-Rt/L
	Black tea Earl Grey, lemon/Liquid	Direct (Bottle of 330 mL)	Direct, R_t_	D-Bt/L
	Lemon balm, linden, chamomile, lemon/Liquid	Direct (Bottle of 330 mL)	Direct, R_t_	D-LbLC/L

^a^ According to manufacturer recommendations. The label of the studied “teas” indicates medicinal uses including antioxidant effects, slimming, anti-carcinogenic, anti-aging, and nervous system stimulation.

### 3.3. Evaluation of Antioxidant Activity

Antioxidant activity of the individual and mixed samples was evaluated by DPPH radical-scavenging activity, reducing power, β-carotene bleaching inhibition and inhibition of lipid peroxidation using TBARS in brain homogenates. The dilution (DF_50_) correspondent to 50% of antioxidant activity (or 0.5 of absorbance for reducing power) was interpolated from the graphs of antioxidant activity (radical scavenging activity, β-carotene bleaching inhibition and inhibition of lipid peroxidation using thiobarbituric acid reactive substances assays) percentages or absorbance at 690 nm (reducing power assay) as function of sample dilutions. The dilution factor range was defined in order to allow percentages of antioxidant activity from ≈10% to ≈90% (stock-solution and successive dilutions). Trolox was used as standard.

#### 3.3.1. Radical Scavenging Activity

This assay was performed using an ELX800 Microplate Reader (Bio-Tek Instruments, Inc., Winooski, VT, USA). The reaction mixture in each one of the 96-wells consisted of sample solution (30 μL) and methanolic solution (270 μL) containing DPPH radicals (6 × 10^−5^ mol/L). The mixture was left to stand for 60 min in the dark. The reduction of the DPPH radical was determined by measuring the absorption at 515 nm [29]. The radical scavenging activity (RSA) was calculated as a percentage of DPPH discolouration using the equation: % RSA = [(A_DPPH_ − A_S_)/A_DPPH_] × 100, where A_S_ is the absorbance of the solution when the sample has been added at a particular level and A_DPPH_ is the absorbance of the DPPH solution.

#### 3.3.2. Reducing Power

This assay was performed using the microplate reader described above. The sample solutions (0.5 mL) were mixed with sodium phosphate buffer (200 mmol/L, pH 6.6, 0.5 mL) and potassium ferricyanide (1% w/v, 0.5 mL). The mixture was incubated at 50 °C for 20 min, and trichloroacetic acid (10% w/v, 0.5 mL) was added. The mixture (0.8 mL) was poured in the 48-wells, as also deionised water (0.8 mL) and ferric chloride (0.1% w/v, 0.16 mL), and the absorbance was measured at 690 nm [29].

#### 3.3.3. β-Carotene Bleaching Inhibition

The antioxidant activity of the samples was evaluated by the β-carotene linoleate model system, as described previously [30]. A solution of β-carotene was prepared by dissolving β-carotene (2 mg) in chloroform (10 mL). Two millilitres of this solution were pipetted into a round-bottom flask. After the chloroform was removed at 40 °C under vacuum, linoleic acid (40 mg), Tween 80 emulsifier (400 mg), and distilled water (100 mL) were added to the flask with vigorous shaking. Aliquots (4.8 mL) of this emulsion were transferred into different test tubes containing different samples dilutions (0.2 mL). The tubes were shaken and incubated at 50 °C in a water bath. As soon as the emulsion was added to each tube, the zero time absorbance was measured at 470 nm (Analytikjena Specord 200 spectrophotometer). β-Carotene bleaching inhibition was calculated using the following equation: (β-carotene content after 2 h of assay/initial β-carotene content) × 100.

#### 3.3.4. Inhibition of Lipid Peroxidation using Thiobarbituric Acid Reactive Substances (TBARS)

Brains were obtained from pig (*Sus scrofa*) of body weight ≈150 Kg, dissected and homogenized with a Polytron in ice-cold Tris-HCl buffer (20 mM, pH 7.4) to produce a 1:2 (w/v) brain tissue homogenate which was centrifuged at 3,000 *g* for 10 min. An aliquot (0.1 mL) of the supernatant was incubated with the samples dilutions (0.2 mL) in the presence of FeSO_4_ (10 μM; 0.1 mL) and ascorbic acid (0.1 mM; 0.1 mL) at 37 °C for 1 h. The reaction was stopped by the addition of trichloroacetic acid (28% w/v, 0.5 mL), followed by thiobarbituric acid (TBA, 2%, w/v, 0.38 mL), and the mixture was then heated at 80 °C for 20 min. After centrifugation at 3,000 *g* for 10 min to remove the precipitated protein, the colour intensity of the malondialdehyde (MDA)-TBA complex in the supernatant was measured by its absorbance at 532 nm [30]. The inhibition ratio (%) was calculated using the following formula: Inhibition ratio (%) = [(A − B)/A] × 100%, where A and B were the absorbance of the control and the compound solution, respectively.

### 3.4. Biomolecules with Antioxidant Activity

#### 3.4.1. Phenolics

The stock-solution (250 μL) was mixed with HCl 0.1% in 95% ethanol (250 μL) and HCl 2% (4,550 μL). After 15 min the absorbance was measured at 280, 320, 360, and 520 nm. The absorbance at 280 nm was used to estimate total phenolic content (mg of chlorogenic acid equivalents (ClAE) per mL of stock solution), A_320nm_ was used to estimate tartaric esters content (mg of caffeic acid equivalents (CAE) per mL of stock solution), A_360nm_ was used to estimate flavonols (mg of quercetin equivalents (QE) per mL of stock solution), and A_520nm_ was used to estimate anthocyanins (mg of malvidin 3-glucoside equivalents (ME) per mL of stock solution) [31]. For flavonoids quantification, the stock-solution (0.5 mL) was mixed with distilled water (2 mL) and NaNO_2_ solution (5%, 0.15 mL). After 6 min, AlCl_3_ solution (10%, 0.15 mL) was added and allowed to stand for a further 6 min. NaOH solution (4%, 2 mL) was added to the mixture, followed by distilled water until a final volume of 5 mL. The mixture was properly mixed and allowed to stand for 15 min. The intensity of pink colour was measured at 510 nm [32]. (+)-Catechin was used to calculate the standard curve and the results were expressed as mg of (+)-catechin equivalents (CE) per mL of stock-solution.

#### 3.4.2. Ascorbic Acid

After lyophilisation (Ly-8-FM-ULE, Snijders, Holland) of the stock-solution, the sample (150 mg) was extracted with metaphosphoric acid (1%, 10 mL) for 45 min at room temperature and filtered through Whatman N° 4 filter paper. The filtrate (1 mL) was mixed with 2,6-dichloroindophenol (9 mL) and the absorbance was measured after 30 min at 515 nm [29]. Content of ascorbic acid was calculated on the basis of the calibration curve of authentic L-ascorbic acid and the results were expressed as mg of ascorbic acid per mL of stock-solution.

### 3.5. Statistical Analysis

All the assays were carried out in triplicate in three different samples of each single formulation and the results were expressed as mean values ± standard deviation (SD). The influence of different “tea” compositions over antioxidant activity and bioactive compounds contents was evaluated using one-way analysis of variance (ANOVA) followed by Tukey’s honestly significant difference *post hoc* test with α = 0.05, coupled with Welch’s statistic. The assumption that the group variances were equal was checked through the Levene’s statistic.

In addition, a linear discriminant analysis (LDA) was used to categorize different “tea” formulations according to their antioxidant capacity, as well as their bioactive compounds contents. A stepwise technique, using the Wilks’ λ method with the usual probabilities of *F* (3.84 to enter and 2.71 to remove), was applied for variable selection. This procedure uses a combination of forward selection and backward elimination procedures, in which the selection of a new variable to be included is preceded by the verification of significance of all previously selected variables [33]. The Wilks’ λ test was applied to verify which canonical discriminant functions were significant. Also a leaving-one-out cross-validation procedure was carried out to assess the model performance. All statistical tests were performed at a 5% significance level. These analysis were performed using SPSS (v. 18.0) program.

## 4. Conclusions

The results showed that either preparation method or formulation influence the antioxidant properties of “teas”. Furthermore, data confirmed and validated the antioxidant benefits indicated in labels. All formulations had capacity to scavenge free radicals such as DPPH, inhibit lipid peroxidation in a β-carotene-linoleate system and inhibit TBARS formation, showing also high reducing power.

Green tea was the most active herbal beverage, but with different behaviours according to the formulation used: liquid extract (D-Gt/E) gave the best scavenging effects and reducing power, the liquid drink with lemon (D-GtL/L) showed the highest β-carotene bleaching inhibition, and the bag infusion (I-Gt/B) showed the strongest TBARS inhibition. Nevertheless, in view of the DF_50_ values, some suggested preparation methods should be adjusted in order to obtain lower concentrations. Despite some critical concerns like the bioavailability and stability of ingested compounds, as well as their most common derived metabolites, this dietary adjustment may prevent eventual pro-oxidant effects.

The quantitative profile of biomolecules with antioxidant potential obtained was similar for all the studied formulations, with ascorbic acid as the main compound, followed by flavonoids. Even so, phenolics (I-GtH/B), flavonols (I-Rt/B), tartaric esters (I-GtH/B) and ascorbic acid (I-Gt/B) were higher in infusions.

The increasing interest for health and wellness boosts the emergence of products containing functional molecules. In view of the results herein obtained new “tea” formulations might be assayed, maintaining their recognized antioxidant activity. From the perspective of the consumer interest in natural products containing bioactive components, these results might be useful for the comprehension of the real effectiveness of bioactive foods, regarding their functional purposes. Furthermore, the application of excessive amounts of antioxidants in everyday diet could lead to increased susceptibility to age-related diseases [34], enhancing the need of defining the most appropriate formulations.
